# Wearable Devices Combined with Artificial Intelligence—A Future Technology for Atrial Fibrillation Detection?

**DOI:** 10.3390/s22228588

**Published:** 2022-11-08

**Authors:** Marko Mäkynen, G. Andre Ng, Xin Li, Fernando S. Schlindwein

**Affiliations:** 1 School of Engineering, University of Leicester, Leicester LE1 7RH, UK; mm915@leicester.ac.uk (M.M.); xl251@leicester.ac.uk (X.L.); 2 National Institute for Health Research Leicester Cardiovascular Biomedical Research Centre, Glenfield Hospital, Leicester LE5 4PW, UK; gan1@leicester.ac.uk; 3 Department of Cardiovascular Sciences, University of Leicester, UK, Leicester LE1 7RH, UK

**Keywords:** wearable devices, artificial intelligence, atrial fibrillation

## Abstract

Atrial fibrillation (AF) is the most common cardiac arrhythmia in the world. The arrhythmia and methods developed to cure it have been studied for several decades. However, professionals worldwide are still working to improve treatment quality. One novel technology that can be useful is a wearable device. The two most used recordings from these devices are photoplethysmogram (PPG) and electrocardiogram (ECG) signals. As the price lowers, these devices will become significant technology to increase sensitivity, for monitoring and for treatment quality support. This is important as AF can be challenging to detect in advance, especially during home monitoring. Modern artificial intelligence (AI) has the potential to respond to this challenge. AI has already achieved state of the art results in many applications, including bioengineering. In this perspective, we discuss wearable devices combined with AI for AF detection, an approach that enables a new era of possibilities for the future.

## 1. Introduction

Atrial fibrillation (AF) has become a global problem [1]. Despite all efforts to tackle it, professionals are still eager to discover new groundbreaking solutions and significant improvements to existing ones. The detection of AF can play a crucial role in treatment quality. For example, early detection of the arrhythmia helps to prevent severe consequences. Therefore, technologies that can monitor the patient at home and provide information related to cardiac health are useful.

Cardiac monitoring can be a prolonged process. Moreover, it is costly, also causing treatment quality challenges. Early detection can help prevent, for example, embolic stroke [2]. Typically, the electrocardiogram (ECG) is used for short- or long-term observation. The traditional method needs many recordings for diagnosis. Alternatively, photoplethysmogram (PPG) recordings are sometimes used as preliminary information [3].

Artificial intelligence (AI) has become an interesting tool to use in many challenging tasks. As bigger annotated datasets have become available, the performance of AI-based techniques has improved significantly. Furthermore, improved optimization methods and increased computational power have supported the progress, along with novel models [4]. Many applications seeking breakthroughs are being increasingly combined with AI.

This perspective is organized as follows: section one is about different recording devices, section two is about AI, and the third section includes a discussion. In the first section, the focus is on wearable devices, mainly for private use. The second section reviews the modern AI field. The third is about the point of view concerning wearable devices and AI combinations (for AF).

### 1.1. Wearable Devices

#### 1.1.1. ECG and PPG

The two most common recording techniques for arrhythmias in smart devices are the ECG and PPG. The ECG is based on the heart’s electrical activity, measured from the body surface [5]. Starting from the SA node, the electrical activation progresses through the atria to the AV node and then through the ventricles before the heart tissue returns to the rest state. These phases are shown in the ECG as the P wave, the QRS complex, and the T wave (followed by U wave). The P wave concerns atrial contraction, the QRS complex relates to ventricular activation, and the T wave corresponds to the return to resting. One healthy heartbeat consists of all of these, but in the case of AF, the P wave is replaced by a ripple F wave, and the RR intervals differ irregularly.

**Figure 1:**
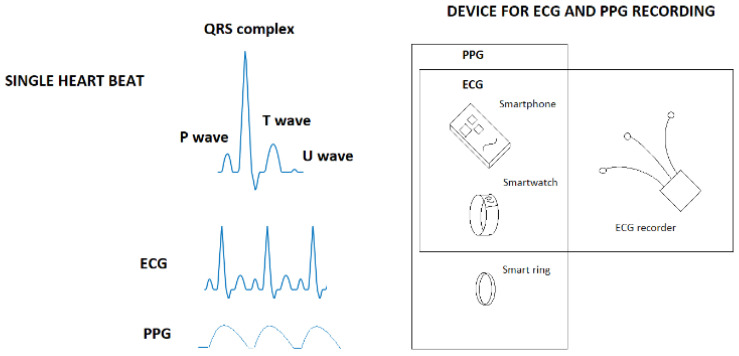
Wearable devices and recording options.

The PPG signal records the blood flow (volume caused by pressure) from the skin by measuring the reflecting light [6]. It is related to R peaks time stamps (RR intervals) in the ECG. Figure 1 shows an ideal ECG (in a sinus rhythm) and PPG signal, along with categories of devices that contain ECG and PPG recordings.

#### 1.1.2. Smart Devices

Smart devices present the most emerging technology for the future. Three current main devices are smartphones, smartwatches, and smart rings. Smartphones are not exactly wearable devices, but people carry them on their person, which is why they are also mentioned. Smartphones are the most used smart devices so far. Some have a PPG recording function by placing the camera on a fingertip [7]. Furthermore, these devices have an ECG patch option [8]. Smartwatches are mainly used for PPG recording, but an ECG possibility exists [9]. Watches are also popular among smart devices and can be used for short and long recordings; therefore, they offer the potential for future applications. The smart ring is the newest technology in this area. It has all the benefits required for recording [10]. Rings can be used for multiple purposes [11]. However, they are still mostly a single-task device. Because of their user-friendly and simple interface, these devices constitute potential future technology.

#### 1.1.3. ECG Recorders

ECG recordings can be performed using different amounts of electrodes. The more electrodes, the higher the resolution of the information produced. A single electrode recording can be taken from different places of the body, but 12-lead ECG are collected from specific spots. Both wired and wireless recording devices are available. Some of the wired devices are similar to smartphones in the sense that they are not actually wearable. The ECG recording downside is uncomfortable patches. 

### 1.2. Artificial Intelligence

AI, especially machine learning, has developed fast and has gained popularity recently. It has contributed to many applications, including medical applications [12]. For example, image classification and speech recognition are two of the most known contributions [4]. In recent years, it has become one of the most focused research fields. AI is not new technology, but many groundbreaking contributions are relatively new. Therefore, more significant outcomes can be expected. AI is a wide area of algorithms and models that consists of sub-branch machine learning, including deep learning [13]. The training is implemented using supervised, unsupervised, or reinforcement learning, and output prediction is either for classification or regression tasks. The machine learning field in this preview is divided into three subfields: traditional machine learning, deep learning, and deep reinforcement learning. 

Supervised learning is based on labeled data to train the model. The classification task is a good example of supervised learning. Unsupervised learning, in contrast, forms an unknown pattern directly from the data. The reinforcement learning in this preview is taken as deep reinforcement learning (DRL). DRL is the latest significant novel contribution to medical applications [14]. It is drawn from animal behavior psychology, which also relates to human brain function [15]. Based on the action, state, and reward relationship as agent acting in the environment, the goal is to maximize the reward by optimizing the policy, which defines the action the agent chooses. The policy in DRL is deep neural network (DNN), and the policy update is achieved by updating DNN weights towards minimizing some error function, as in a supervised learning fashion.

Traditional machine learning includes models more restricted in structure than deep learning. Nevertheless, these can be robust. For example, a decision tree is suitable as a simple model, but its accuracy can decrease when it becomes too complex [16]. The benefit of these models is a low computational requirement. However, it does not always provide an initially optimal outcome for every application, as deep learning models do [17].

Deep neural networks (DNN) have gained a lot of attention in recent years. A DNN mimics human brain functionality [18] and is, in many cases, a simulation tool for brain research [19]. DNNs have produced state-of-the-art results in AF detection [20]. These models’ benefits are the optimal use of large datasets and the possibility of deep structure [4]. Nevertheless, many choices exist to optimize the final model.

DNN has become a popular tool because it can be trained using raw data with state-of-the-art results. From this perspective, DNN has surpassed the performance of traditional machine learning models in some applications. However, both have their own benefits, which should be considered when designing one for a task.

Machine learning has many tasks with AF to apply in addition to detection [21], such as risk assessment in real time and AF management. One significant task to tackle with arrhythmias using DNN is detection using embedded or wearable devices [22]. It would have a significant impact on the future if these devices integrated with DNN were part of diagnosing and treating AF. The DRL can also contribute to the future by consolidating the treatment; for example, the selection of ablation areas for curing AF using catheter ablation can potentially be improved by DRL [23]. 

## 2. Discussion

Wearable devices can play a significant role in AF detection and in supporting treatments in the future. Furthermore, AI will provide a tool to answer demand in the long run. This combination is already under study [3] and is likely to take a more substantial role in novel approaches. There are many benefits to combining these two technologies. Perhaps one of the significant perspectives is personalized treatment [24]. A classifier whose performance is on a par with the level of cardiologists will make significant differences in the future [25].

AF and its details are highly diverse in terms of its electrophysiology. For example, cardiologists must consider multiple factors outside of the AF knowledge and cardiological details of AF before the diagnosis [26]. This procedure includes checking which drugs the patient is already using and possible symptoms to avoid a false connection to AF. Furthermore, the doctor has to match the AF-related symptoms when considering diagnosis. Eventually, the gold standard ECG (12-lead, possibly) is analyzed to confirm the diagnosis. However, the ECG analysis is not easy because it can contain a lot of noise, and many recordings are needed for a robust diagnosis. This is where AI can facilitate the process when considered in combination with wearable devices. 

In AF detection, early detection plays a vital role. Embolic stroke is a severe problem with AF, and high-quality treatment demands early detection [2]. In many cases, the patient does not consult a cardiologist before clear symptoms arise. Therefore, home devices would be useful for early detection. These would mostly be smart devices. Combined with AI, these devices are suitable for the task. With the help of an AI, early-stage detection and management of AF can help preventing further progress of the disease and further atrial damage. Patients with paroxysmal (early) AF can be more easily cured than those with longer-term (persistent or permanent) AF [27]. Early detection of AF events would help improve treatment quality [28].

Machine learning provides a potential tool to respond to challenges with AF detection. Using PPG and accelerator meter recordings, AF can be detected by peak detections after processing to remove motion and noise artifacts [29]. The detection accuracy is reinforced via premature contraction clearance. This method (or similar ones) might require strong signal processing and background knowledge. Another method that has the same aspects is an entropy-based AF detector [30]. The more traditional machine learning models used for detection are, for example, support vector machine with feature vector input [31], decision tree with multilevel features input [32], and random forest with PPG, inter-pulse intervals, and accelerometer features input [33]. Furthermore, k-nearest neighbor and discriminant analysis are also used based on feature inputs [34]. The common factor for these models is feature dependency, and they frequently require significant background knowledge. These are still suitable for low-computational power devices. In addition to these models, stacking classifier, extreme gradient boosting, and adaptive boosting provides potential in AF detection [35]. The factors related to these are the same as for traditional machine learning models, also with stacking classifier if those models are used. Nevertheless, DNN has recently gained more attention [21]. For example, Residual Neural Network can detect AF using RR intervals as inputs [20], and bidirectional long short-term memory network produces significant results on filtered short ECG segment [36]. However, inference (reasoning) behind the DNN prediction would be desired [37]. Therefore, DNN structure is established to produce information about ECG waveform using bidirectional recurrent neural networks combined with an attention model [38] and a diverse deep learning model [39]. DNN’s benefit is its usefulness for end-to-end classifiers using raw data [2]. That has raised significant interest with notable results, but further work is needed to confirm the validation [40]. However, usually these models require significant computational power, which limits their use to small smart devices.

Machine learning use is not problem-free with biological data. There are a couple of fundamental dilemmas with AF. One such challenge is good-quality data gathering [28]. Another challenge is availability of a comprehensive dataset, without which the machine learning model might not become a good enough generalizer [41]. Furthermore, the quality of recordings significantly affects the final outcome [28]. AI researchers are busy developing solutions to these challenges. For example, artificial data generators could address some of these problems [42]. Furthermore, DRL does provide potential for the future.

One significant perspective is DNN development work in the future. DNN suitable for smart devices with far less computational power than laptops, for example, is a considerably attractive perspective. If a wearable device can inform the user of AF signals (for the first time, before symptoms arise), it could become cutting-edge technology for improving treatment quality and lowering costs. Furthermore, privacy, which is very important to many people, could be addressed by keeping information/data in the device only.

The future may bring new wearable devices that record useful data; it could be ECG, PPG, or something else entirely. However, the concept is most likely the same as AI predicting meaningful information. Finally, AI development in the future provides prospects for further innovations [43].
