# Correction: Mäkynen et al. Wearable Devices Combined with Artificial Intelligence—A Future Technology for Atrial Fibrillation Detection? *Sensors* 2022, *22*, 8588

**DOI:** 10.3390/s23187972

**Published:** 2023-09-19

**Authors:** Marko Mäkynen, G. Andre Ng, Xin Li, Fernando S. Schlindwein

**Affiliations:** 1 School of Engineering, University of Leicester, Leicester LE1 7RH, UK; mm915@leicester.ac.uk (M.M.); xl251@leicester.ac.uk (X.L.); 2 National Institute for Health Research Leicester Cardiovascular Biomedical Research Centre, Glenfield Hospital, Leicester LE5 4PW, UK; gan1@leicester.ac.uk; 3 Department of Cardiovascular Sciences, University of Leicester, UK, Leicester LE1 7RH, UK

The authors wish to add two authors to the original paper [1].

The authors are requesting a change of authorship for this paper: The paper was published with just Marko Mäkynen and Fernando S. Schlindwein as authors when it should have listed all four authors (Marko Mäkynen, G. Andre Ng, Xin Li and Fernando S. Schlindwein).

The relevant manuscript contributors are not fully listed. After confirming all authors’ detailed contributions. It was decided to correct the authorship to appropriately list the significant contributions to the intellectual content of the manuscript.

The authors apologize for any inconvenience caused and state that the scientific conclusions are unaffected. This correction was approved by the academic editor. The original publication has also been updated.
